# Prior Ustekinumab Exposure and Ulcerative Colitis Are Associated with Suboptimal Trough Levels in Patients Receiving Advanced-Line Vedolizumab or Ustekinumab for Inflammatory Bowel Disease: A Cross-Sectional Cohort Study

**DOI:** 10.3390/biomedicines14102331

**Published:** 2026-10-14

**Authors:** Petra Nagypál, Krisztián Kovács, Barna Vásárhelyi, Antal Dezsőfi-Gottl, Pál Miheller, Ákos Iliás, Nóra Judit Béres, Anna Balogh, Bence Prehoda, Luca Tóbi, Attila József Szabó, Áron Cseh

**Affiliations:** 1 Paediatric Centre, Semmelweis University, 1083 Budapest, Hungary; nagypal.petra@stud.semmelweis.hu (P.N.); dezsofi.antal@semmelweis.hu (A.D.-G.); beres.nora@semmelweis.hu (N.J.B.); annabalogh94@gmail.com (A.B.); prehoda.bence@gmail.com (B.P.); tobiluca02@gmail.com (L.T.); szabo.attila@semmelweis.hu (A.J.S.); 2 Department of Laboratory Medicine, Semmelweis University, 1089 Budapest, Hungary; kovacs.krisztian1@med.semmelweis-univ.hu (K.K.); vasarhelyi.barna@med.semmelweis-univ.hu (B.V.); 3 Department of Surgery, Transplantation and Gastroenterology, Semmelweis University, 1082 Budapest, Hungary; pmiheller@gmail.com; 4 Department of Internal Medicine and Oncology, Semmelweis University, 1083 Budapest, Hungary; driliasakos@gmail.com

**Keywords:** inflammatory bowel disease, therapeutic drug monitoring, predictive factors, ustekinumab, vedolizumab, monoclonal antibody

## Abstract

**Background**: Ustekinumab (UST) and vedolizumab (VDZ) are established therapies for Crohn’s disease (CD) and ulcerative colitis (UC), including in patients with prior tumour necrosis factor-alpha inhibitor (anti-TNF) failure. Trough levels (TRLs) and antidrug antibodies (ADAs) are among several factors associated with therapeutic response. The aim of this study was to retrospectively identify factors associated with suboptimal TRL and ADA presence in a large, multicentre, cross-sectional cohort. **Methods**: TRLs and ADAs for VDZ and UST were measured routinely during therapy. A Cox proportional hazards model was used to identify clinical and treatment-related factors associated with suboptimal TRLs. **Results**: A total of 184 patients (78 children and 106 adults) with inflammatory bowel disease (IBD) were included; 104 and 80 were treated with UST and VDZ, respectively. TRLs were measured as part of routine clinical care between 2022 and 2025. Of the 184 patients, 80 (43.48%) had previously received both infliximab and adalimumab. Overall, 103 patients (55.98%) had received at least two advanced therapies (infliximab, adalimumab, VDZ, UST or a small-molecule inhibitor (SMI)) before the current biologic therapy. In exploratory Cox analyses, prior UST exposure (HR = 4.13, 95% CI = 1.58–10.81, *p* = 0.004) and UC subtype (HR = 2.83, 95% CI = 1.39–5.76, *p* = 0.004) were associated with a higher hazard of suboptimal TRL, whereas longer duration of prior biologic therapy (HR = 0.89, 95% CI = 0.82–0.96, *p* = 0.003) and current VDZ treatment (HR = 0.36, 95% CI = 0.16–0.82, *p* = 0.014) were associated with a lower hazard. In a cross-sectional logistic model accounting for time on therapy, only longer duration of prior biologic therapy remained significantly associated with suboptimal TRL. Prior UST exposure was associated with ADA presence in the cross-sectional model, although the estimate was imprecise and sensitive to model specification. **Conclusions**: Prior UST exposure, UC subtype and current VDZ therapy were associated with suboptimal TRLs in exploratory Cox analyses; however, these associations were not reproduced in the cross-sectional logistic model and were sensitive to adjustment for inflammatory burden. Longer duration of prior biologic therapy remained associated with lower odds of suboptimal TRL in the cross-sectional analysis. The observed association between prior UST exposure and ADA presence was imprecise and model-dependent. Given the study’s retrospective design and limited sample size, these findings should be considered preliminary and require validation in larger prospective cohorts.

## 1. Introduction

Inflammatory bowel disease (IBD) is a chronic, immune-mediated, relapsing illness comprising three subtypes: Crohn’s disease (CD), ulcerative colitis (UC), and IBD unclassified (IBD-U) [1].

The primary treatment goals in IBD are to induce clinical response, achieve remission, improve quality of life, and, importantly, achieve mucosal healing [2,3].

IBD patients who do not respond to conventional therapies (exclusive enteral nutrition, corticosteroids, aminosalicylates, immunomodulators or combinations) frequently require biologic therapy. TNF-alpha inhibitors have traditionally represented the predominant first-line biologic therapy in IBD and were used as the initial biologic in most patients in our cohort, although contemporary treatment selection is increasingly individualised according to disease phenotype and patient characteristics. However, approximately one in three patients experiences primary nonresponse (PNR) and a further half experience loss of response (LOR) over time. In such cases, switching to an alternative mechanism of action, such as vedolizumab (VDZ) or ustekinumab (UST), represents an established therapeutic strategy [2,3,4,5,6,7,8].

Therapeutic drug monitoring (TDM) based on trough levels (TRLs) and antidrug antibody (ADA) levels supports clinicians in optimising treatment with biologic agents. The relationship between VDZ or UST TRLs and therapeutic response has not been clearly established, although several studies have shown that higher TRLs are associated with mucosal healing and favourable endoscopic outcomes. When interpreted alongside biomarkers, clinical findings, and the overall clinical context, TDM can improve clinical decision-making [9,10,11,12,13].

Patients treated with TNF-alpha inhibitors frequently develop ADAs, which may be associated with LOR [14].

ADAs are reported in 2–20% of patients receiving VDZ. During the maintenance phase, this rate decreases to approximately 3%. Most ADAs during VDZ treatment are transient; only approximately 1% are persistent. Therefore, in most cases, they do not require treatment modification, nor do they consistently correlate with clinical outcomes, suggesting that immunogenicity may not substantially impact VDZ treatment response [15,16,17,18].

In UST-treated patients, the cumulative incidence of ADAs by week 52 is even lower, occurring in 2–5% of participants in most studies. These ADAs are typically transient, present at low titres (below 1:800), and show no association with clinical remission, efficacy or TRLs. No difference has been observed between patients who received and those who did not receive concomitant immunosuppression, further supporting the limited clinical impact of ADAs in UST-treated patients [19,20,21,22,23].

Immunogenicity is not considered a primary driver of treatment failure with VDZ or UST because most ADAs against advanced-line biologics are transient and clinically inconsequential. Nevertheless, ADA monitoring is recommended, as a small proportion of patients may develop persistent, high-titre ADAs that do affect drug clearance and contribute to LOR [24,25,26].

The aim of this study was to identify factors associated with suboptimal TRLs and ADA presence in patients receiving VDZ or UST.

## 2. Materials and Methods

### 2.1. Patients and Study Design

This retrospective multicentre study was conducted at the Department of Paediatrics, the Department of Internal Medicine and Oncology, and the Department of Surgery, Transplantation and Gastroenterology at Semmelweis University, Budapest, Hungary. A total of 184 patients were included: 78 children and 106 adults. VDZ or UST TRLs were measured between August 2022 and October 2025, and patients initiated VDZ or UST treatment between March 2015 and May 2025. Most patients were in clinical remission or had low disease activity at TDM. Previous exposure to one or more biologic therapies was permitted but was not required before initiation of VDZ or UST. Inclusion criteria were a confirmed diagnosis of IBD and ongoing biologic therapy with VDZ or UST. Patients with IBD-U were excluded.

### 2.2. Ethical Statement

Blood samples were taken for routine clinical care; no additional blood samples were collected for research purposes. Written informed consent was obtained from all participants or from their legal guardians before inclusion. The study was approved by the Ethics Committee of the Medical Research Council, Ministry of Interior (Approval No. 19048-Á2/2018/EKU). All data were de-identified to preserve patient anonymity. Study procedures complied with the principles of the 1964 Declaration of Helsinki and its subsequent amendments.

### 2.3. Data Collection

The dataset contained variables collected at different time points. Variables known before initiation of the current biologic included IBD subtype, disease phenotype, gender, age at disease onset, age at biologic therapy initiation, type of prior biologic therapy (infliximab, adalimumab, VDZ, UST or a small-molecule inhibitor (SMI)), immunosuppressant, corticosteroid and 5-aminosalicylate therapy, duration and number of prior biologic treatments, disease activity indices at switch to current treatment, and the choice of the current biologic. Variables assessed at the TDM visit included age at measurement, concomitant immunosuppressant, corticosteroid and 5-aminosalicylate therapy, laboratory parameters (C-reactive protein (CRP), white blood cell count (WBC), haemoglobin, platelets, and albumin), body mass index (BMI), disease activity indices (PUCAI, PCDAI, CDAI and pMayo), clinical remission or relapse status, and information on biologic dose intensification. Variables occurring during subsequent follow-up included surgery, the number and types of subsequent biologic switches, and treatment duration before switching.

Disease activity was assessed using validated activity indices or, when sufficient data to calculate an activity index were unavailable, explicit documentation of relapse or remission in healthcare records. Adult patients with CD were evaluated using the Crohn’s Disease Activity Index (CDAI); adult UC patients were assessed using the partial Mayo score (pMayo). Paediatric patients were evaluated using the Pediatric Crohn’s Disease Activity Index (PCDAI) or the Pediatric Ulcerative Colitis Activity Index (PUCAI). Patients were classified as being in remission (CDAI < 150, pMayo < 3, PUCAI/PCDAI < 10) or in relapse (CDAI ≥ 150, pMayo ≥ 3, PUCAI/PCDAI ≥ 10). The target TRL and ADA levels were the same for patients in remission and those experiencing relapse.

### 2.4. Drug Regimens

VDZ induction dosing comprises 300 mg administered intravenously at weeks 0, 2, and 6, followed by maintenance therapy every 8 weeks. For patients with reduced response, the dosing interval may be shortened to 4 weeks.

UST induction is weight-based and administered intravenously, followed by 90 mg subcutaneous injections every 8–12 weeks during maintenance. If TRLs are suboptimal, the dosing interval may be reduced to 4 weeks to improve clinical outcomes and sustain remission [27]. Interval shortening was considered in patients with biomarker or endoscopic evidence of active inflammation, with or without active symptoms [6,7]. In patients with LOR, reactive TDM may help distinguish pharmacokinetic from mechanistic treatment failure, although its role with VDZ and UST remains limited. In our study, reactive TDM was used to support treatment optimisation, with suboptimal TRLs in the absence of high-titre ADAs favouring dose intensification.

### 2.5. Therapeutic Drug Monitoring

TDM involves the systematic measurement of drug TRLs and ADAs during biologic treatment. Multiple studies have demonstrated correlations between clinical and endoscopic responses and TRLs [28,29]. TRLs and ADAs were measured from blood samples using the Lisa-Tracker Duo Vedolizumab and Lisa-Tracker Duo Ustekinumab in vitro diagnostic ELISA kits (Biosynex Theradiag, Croissy-Beaubourg, France). Analyses were performed using a DAS APE ELITE ELISA analyser (DAS Instruments, Rome, Italy) according to the manufacturer’s instructions. Internal quality control procedures were applied using patient samples to ensure assay validity and assess within-run reproducibility.

No consensus exists on target TRLs for non-anti-TNF monoclonal antibodies; however, several studies have proposed clinically relevant thresholds. In the present study, the therapeutic thresholds recommended by the assay manufacturer (Theradiag), based on published exposure–response data, were applied: for UST ≥ 3.5 µg/mL post-induction and ≥1 µg/mL during maintenance; for VDZ ≥ 15 µg/mL post-induction and ≥12 µg/mL during maintenance [30]. These cut-offs fall within ranges previously associated with favourable treatment outcomes [28,29,31]. The same therapeutic TRL thresholds were applied to patients receiving intensified and standard maintenance dosing. For post-induction TDM, samples were collected immediately before the first maintenance dose: 8 weeks after intravenous UST induction or 8 weeks after the third VDZ induction dose. For maintenance TDM, samples were collected immediately before the next scheduled maintenance dose.

### 2.6. Statistical Analysis

Continuous variables are reported as medians with interquartile ranges (IQR); categorical variables are reported as counts with percentages. Baseline characteristics were described using descriptive statistics.

Factors associated with suboptimal TRL, ADA presence, and treatment switch were assessed using Cox proportional hazards regression models. For TRL and ADA outcomes, each patient had a single TDM measurement. Therefore, the true time of onset of suboptimal TRL or ADA presence was unknown. The analysis time in the Cox models corresponded to the interval between initiation of the current biologic therapy and TDM sampling rather than to an observed event time. Because these data represent cross-sectional current-status observations, the Cox analyses were considered exploratory and were supplemented by multivariable logistic regression models, with time on therapy included as a covariate. For treatment switch, time was measured from TDM sampling to biologic switch or last follow-up.

The TRL and ADA models included variables known before initiation of the current biologic. Variables measured at the TDM visit or occurring thereafter were not included as predictors in the primary TRL or ADA Cox models. No time-dependent Cox models were fitted; covariates in the primary models were treated as fixed variables. The final TRL and ADA models were specified on clinical grounds before analysis. Candidate variables were excluded a priori for three reasons: substantial missing data, temporal inappropriateness (i.e., measurement at or after the TDM visit), or the limited number of outcome events, which constrained the number of estimable parameters. Variables with substantial missingness included disease activity indices, albumin, and detailed disease localisation and behaviour. Results are reported as hazard ratios (HR) with 95% confidence intervals (CI); model fit was assessed using the likelihood ratio chi-squared statistic.

The proportional hazards assumption was evaluated using Schoenfeld residuals. This assumption requires that the effect of a given factor on the outcome remains constant throughout follow-up; when it is not met, the corresponding hazard ratio should be interpreted as an average effect over the whole follow-up period rather than as a constant effect. Kaplan–Meier curves stratified by prior UST exposure were generated for each outcome. For treatment switching, group differences were assessed using the log-rank test; for TRL and ADA, the curves were treated as exploratory descriptive displays only. All analyses were performed in R (version 4.5.0) using the survival package. A two-sided *p*-value < 0.05 was considered statistically significant. Because the three final multivariable models contained 7, 5, and 7 estimated covariate coefficients, respectively, the resulting 19 hypothesis tests were adjusted jointly using the Benjamini–Hochberg false discovery rate (FDR) procedure. The adjustment was therefore applied to the coefficients actually estimated in the final models rather than to the broader set of candidate variables considered during model development. The FDR adjustment was confined to these 19 prespecified primary-model coefficients; all sensitivity analyses were exploratory and are reported unadjusted. Both unadjusted *p*-values and FDR-adjusted q-values are reported. Missing covariate data were handled by complete-case analysis: the TRL and treatment-switch models included all 184 patients, as every covariate entered into them was fully documented, whereas the ADA model included 122 patients because prior immunosuppressant therapy was documented for only 122 patients; no imputation was performed. The q-value may be interpreted as a *p*-value corrected for the number of tests performed; it controls the expected proportion of false-positive results among the associations declared significant.

Because TRL and ADA status were each determined from a single cross-sectional TDM measurement, and because several covariates were incompletely documented, a set of sensitivity analyses was performed to establish whether the main findings depended on these features of the data rather than reflecting genuine associations. In the Cox models, the analysis time for the TRL and ADA outcomes is the interval between the start of the current biologic and the TDM sample, since this is the only time point at which these two outcomes were observed; if this interval differs systematically between patient groups, it may influence the estimates independently of the outcome itself. Both models were therefore also fitted as multivariable logistic regression models, in which the outcome is the presence or absence of the finding at the time of sampling and the time spent on therapy is entered as an ordinary covariate. Because suboptimal TRL and detectable ADA are not necessarily irreversible states, these measurements were not modelled as interval-censored survival events; agreement between the time-to-detection and cross-sectional frameworks was used to judge the robustness of each association.

Disease activity (relapse/remission status) was unavailable in 24 patients (13%). All three models were refitted in the subgroup with complete activity data to verify that the results were not driven by the omission of these patients. The TRL model was additionally refitted with relapse status and CRP as covariates, since active inflammation may accelerate drug clearance and could therefore account for part of the observed associations.

To examine whether the association between prior UST exposure and the outcome differed according to age group, interaction terms between prior UST exposure and age group were tested. Because only 14 patients had prior UST exposure, these tests had limited power, and a non-significant result indicates the absence of detectable interaction rather than evidence of equivalence. Furthermore, because only 14 patients had prior UST exposure and regression estimates derived from such small subgroups tend to be unstable and exaggerated, the ADA model was refitted using ridge penalisation, a method that shrinks the estimates towards the null value and thus yields more conservative results.

## 3. Results

### 3.1. Descriptive Statistics

Baseline clinical and treatment characteristics are summarised in Table 1. A total of 184 patients were included: 78 children (42.4%) and 106 adults (57.6%). The cohort comprised 98 female and 86 male patients with a median age at disease onset of 19.2 years (IQR 13.1–27.7). Of these, 74 patients (29 children, 45 adults) had UC, and 110 patients (49 children, 61 adults) had CD. During the study, 104 patients received UST and 80 received VDZ.

Most patients were biologic-experienced; only five patients (2.72%) were biologic-naïve. Nearly half of the patients (43.48%) had previously received both infliximab and adalimumab as separate treatment courses before switching to VDZ or UST. In the overall cohort, 44 patients (23.91%) had received one or two anti-TNF agents and a non-anti-TNF biologic agent, either VDZ or UST, as separate treatment courses in varying sequences. Seven patients had received an SMI. Overall, 103 of 184 patients (55.98%) had received at least two biologic agents during their treatment history.

The median time from diagnosis to first biologic therapy was 3.83 years (IQR 0.87–9.0), and the median interval from the first biologic to the initiation of the measured biologic was 2.67 years (IQR 0.92–5.85). TDM measurements were obtained at a median of 50.93 weeks (IQR 22.79–116.32) after treatment initiation. The median time from diagnosis to measurement was 9.83 years (IQR 5.26–15.67). At the time of TDM, 52.18% of the cohort was in remission (43 VDZ, 53 UST) and 34.78% was in relapse (28 VDZ, 36 UST). At the time of TDM, 30 patients had moderate or severe disease activity, including 21 patients with UC and 9 with CD. Among these patients, 18 were receiving VDZ and 12 were receiving UST treatment. In 13% of patients (24/184), disease activity could not be calculated owing to incomplete documentation.

**Figure 1:**
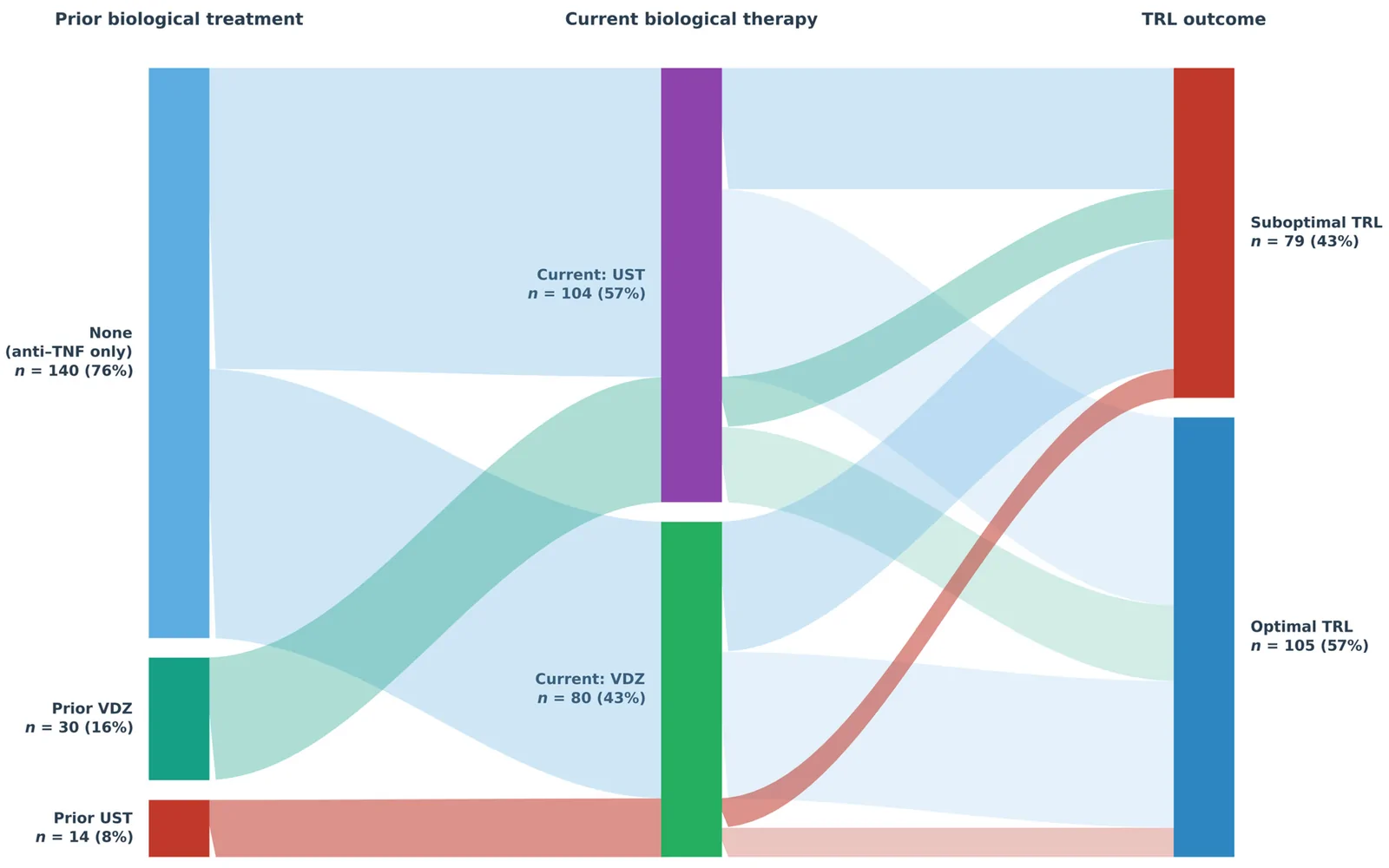
Patient journey from prior biologic treatment history through current drug choice to TRL outcome. Stream widths are proportional to patient counts; colours track patients’ prior biologic treatment history throughout the journey. The prior-UST cohort (red, *n* = 14, 8%) is shown separately to illustrate its distribution through subsequent treatment and across optimal and suboptimal TRL outcomes. Abbreviations: anti-TNF: tumour necrosis factor-alpha, TRL: trough level, UST: ustekinumab, VDZ: vedolizumab.

The composition of the cohort is summarised in Figure 1, which traces each patient through three sequential stages: prior treatment history, the drug chosen for the current treatment, and the resulting TRL status.

A Cox proportional hazards model was constructed to identify factors associated with a higher or lower hazard of suboptimal TRL (*N* = 184, events = 79, χ^2^(7) = 22.51, *p* = 0.002). The multivariable model identified two factors associated with a higher hazard of the outcome and two associated with a lower hazard.

### 3.2. Factors Associated with a Higher Hazard of Suboptimal Trough Level

The strongest factor associated with suboptimal TRL was prior UST treatment, corresponding to an approximately fourfold higher hazard (HR = 4.13, 95% CI = 1.58–10.81, *p* = 0.004). As most patients had also received prior anti-TNF therapy, this finding implicitly encompasses prior anti-TNF exposure; however, no association was observed for prior VDZ exposure (HR = 0.65, 95% CI = 0.30–1.41, *p* = 0.274) or prior anti-TNF therapy alone.

UC subtype was associated with an approximately threefold higher hazard of suboptimal TRL (HR = 2.83, 95% CI = 1.39–5.76, *p* = 0.004). Both associations remained significant after FDR adjustment (prior UST, q = 0.015; UC subtype, q = 0.015).

### 3.3. Factors Associated with a Lower Hazard of Suboptimal Trough Level

Long cumulative exposure to prior advanced therapies, including anti-TNF agents, VDZ, UST and SMIs, was associated with a modest reduction in the hazard of suboptimal TRL (HR = 0.89, 95% CI = 0.82–0.96, *p* = 0.003). Each additional year of prior treatment exposure was associated with an 11% lower hazard of suboptimal TRL.

**Figure 2:**
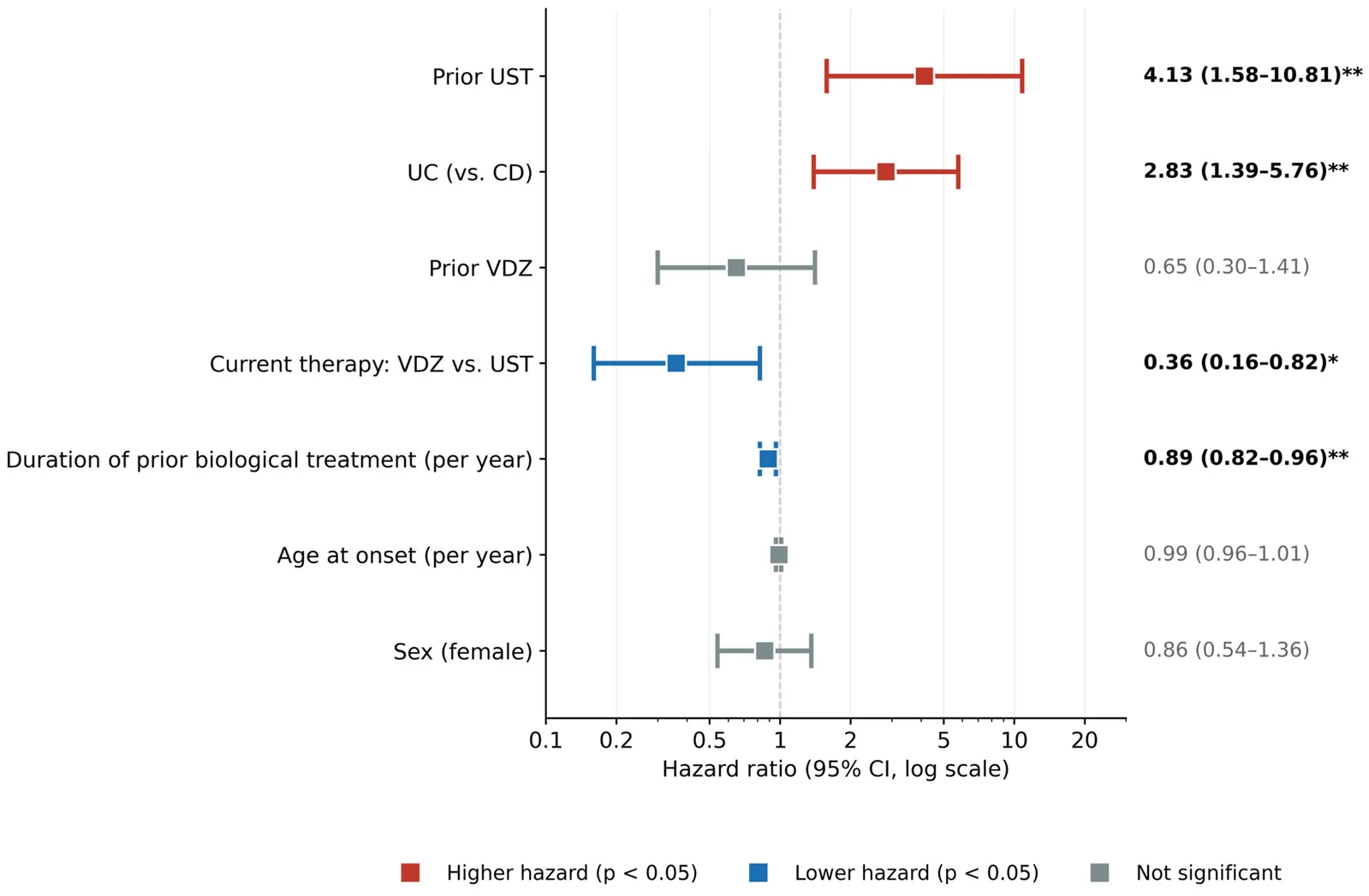
Forest plot of the Cox proportional-hazards model for suboptimal trough levels (TRL) (*N* = 184, events = 79, χ^2^(7) = 22.51, *p* = 0.002). Hazard ratios (HR) are shown as squares with 95% confidence intervals (horizontal bars) on a log scale. Red squares indicate factors significantly associated with a higher hazard (HR > 1, *p* < 0.05); blue squares indicate factors significantly associated with a lower hazard (HR < 1, *p* < 0.05); grey squares indicate non-significant factors. Numeric labels show HR (95% CI); * *p* < 0.05, ** *p* < 0.01. The dashed vertical line marks HR = 1 (no effect). Reference categories: CD (vs. UC), prior adalimumab/infliximab (vs. prior VDZ or UST), UST as the current therapy (vs. VDZ). Abbreviations: CD: Crohn’s disease, CI: confidence interval, UC: ulcerative colitis, UST: ustekinumab, VDZ: vedolizumab.

VDZ treatment was also identified as a factor associated with a lower hazard of suboptimal TRL in patients without prior UST exposure (HR = 0.36, 95% CI = 0.16–0.82, *p* = 0.014), representing an approximately two-thirds lower hazard. Among patients with prior UST exposure, all subsequently received VDZ; therefore, the association of current VDZ versus UST therapy could not be evaluated separately within this subgroup. Both associations with a lower hazard of suboptimal TRL remained significant after FDR adjustment (duration of prior biologic therapy, q = 0.015; current VDZ therapy without prior UST exposure, q = 0.044). The complete set of hazard ratios is presented as a forest plot in Figure 2. Numerical estimates for every factor entered into the three Cox models, including hazard ratios, 95% confidence intervals, unadjusted *p*-values, FDR-adjusted q-values and reference categories, are summarised in Table 2.

### 3.4. Antidrug Antibody Presence

**Figure 3:**
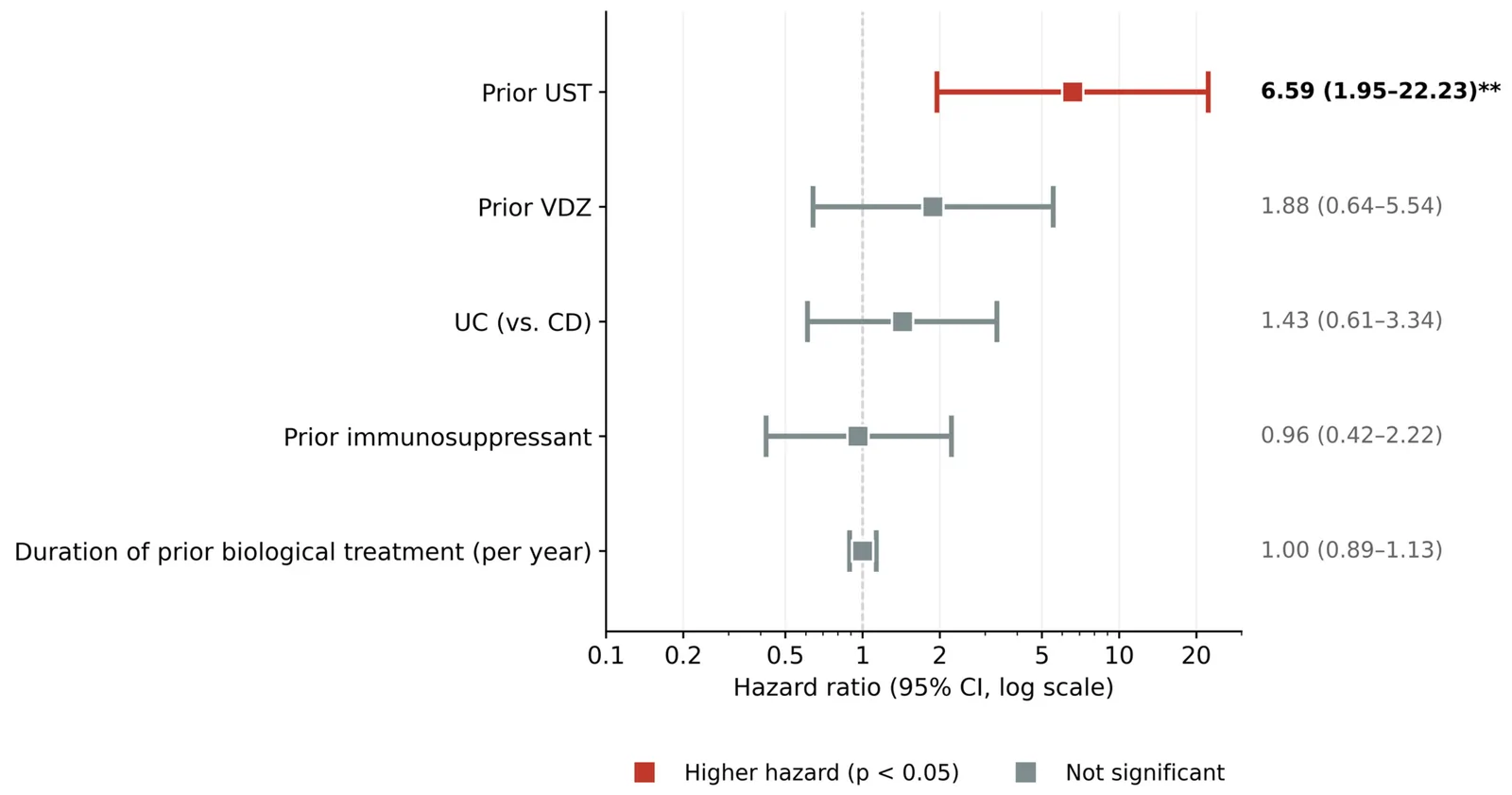
Forest plot of the Cox proportional-hazards model for ADA presence (*N* = 122, events = 27). Hazard ratios are shown as squares with 95% confidence intervals on a log scale. Red squares indicate factors significantly associated with a higher hazard (HR > 1, *p* < 0.05); grey squares indicate non-significant factors. Numeric labels show HR (95% CI); ** *p* < 0.01. The dashed vertical line marks HR = 1 (no effect).

Associations between candidate variables and ADA presence were evaluated using Cox proportional hazards regression. Among all variables tested, only prior UST treatment was significantly associated with ADA presence, corresponding to a more than sixfold higher hazard (HR = 6.59, 95% CI = 1.95–22.23, *p* = 0.002). IBD subtype, prior VDZ treatment, prior immunosuppressive therapy, and duration of prior biologic therapy were not significantly associated with ADA presence when other variables were held constant. The hazard ratios for all variables entered in the ADA model are presented in Figure 3. The association with prior UST treatment remained significant after FDR adjustment (q = 0.015).

### 3.5. Treatment Change During Follow-Up

**Figure 4:**
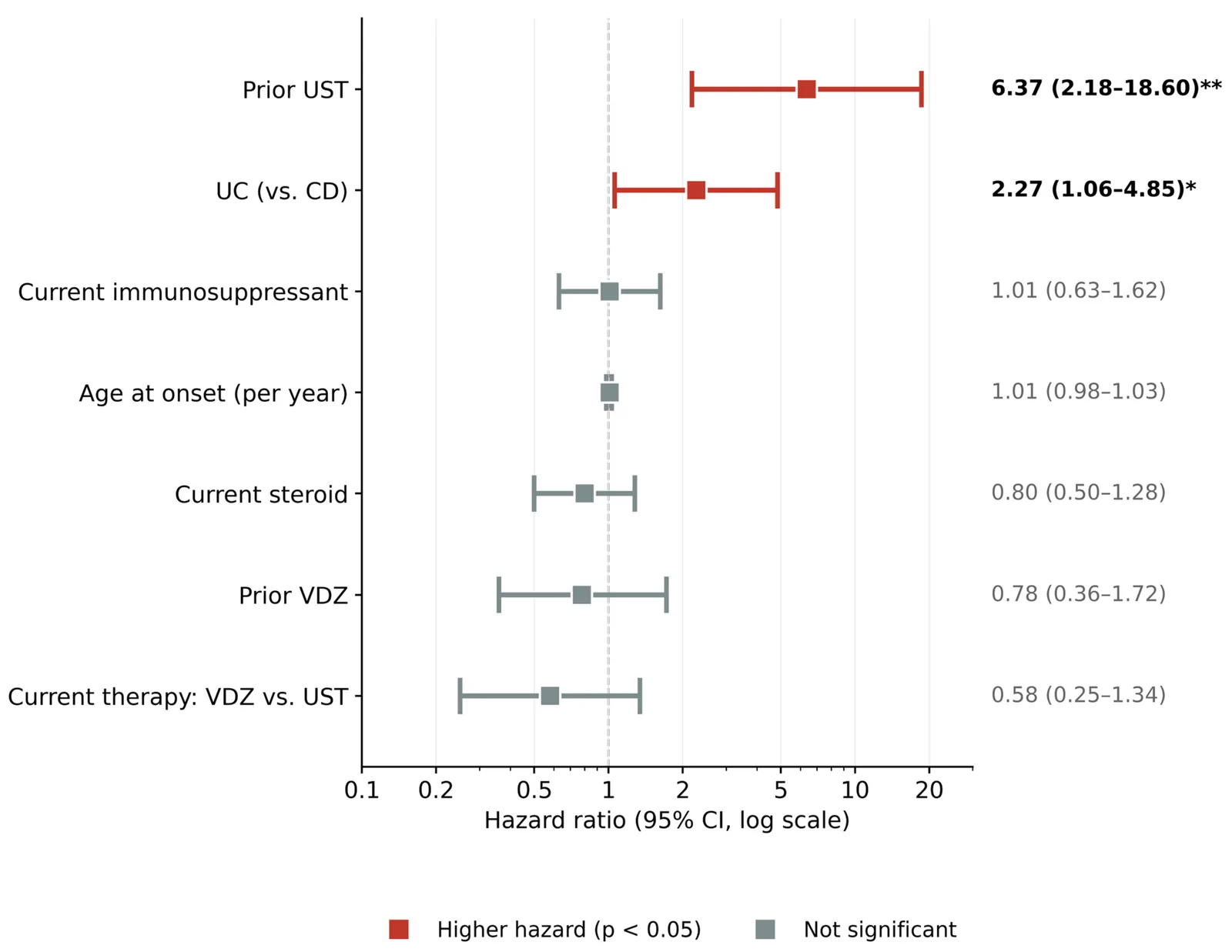
Forest plot of the Cox proportional-hazards model for treatment switch during follow-up (*N* = 184, events = 77). Hazard ratios are shown as squares with 95% confidence intervals on a log scale. Red squares indicate factors significantly associated with a higher hazard (HR > 1, *p* < 0.05); grey squares indicate non-significant factors. Numeric labels show HR (95% CI); * *p* < 0.05; ** *p* < 0.01. The dashed vertical line marks HR = 1 (no effect). Reference categories: CD (vs. UC), prior adalimumab/infliximab, UST as the current therapy, no concomitant immunosuppressant or steroid.

After TDM, the median follow-up duration was 20.04 months (IQR 15.29–24.86). In the treatment-switch model (N = 184, events = 77), the factors most strongly associated with switching were UC subtype (HR = 2.27, 95% CI = 1.06–4.85, *p* = 0.034) and prior UST treatment (HR = 6.37, 95% CI = 2.18–18.60, *p* < 0.001). Overall, 41.85% (77/184) of patients required a biologic switch: 34 receiving UST and 43 receiving VDZ. The recorded reasons for treatment switching were unknown in 56 of 77 patients, pharmacokinetic failure in 3, pharmacodynamic failure in 5, immunological failure in 9, and side effects in 4. Because approximately one quarter of the events were unknown, a specific sensitivity analysis could not be performed reliably. Among patients who switched treatment, 35% (27/77) underwent at least two switches. Two patients decided not to receive any further biologic therapy. The hazard ratios for this model are shown in Figure 4. After FDR adjustment, the association with prior UST treatment remained significant (q = 0.015), whereas the association with UC subtype no longer reached the significance threshold (q = 0.092).

**Figure 5:**
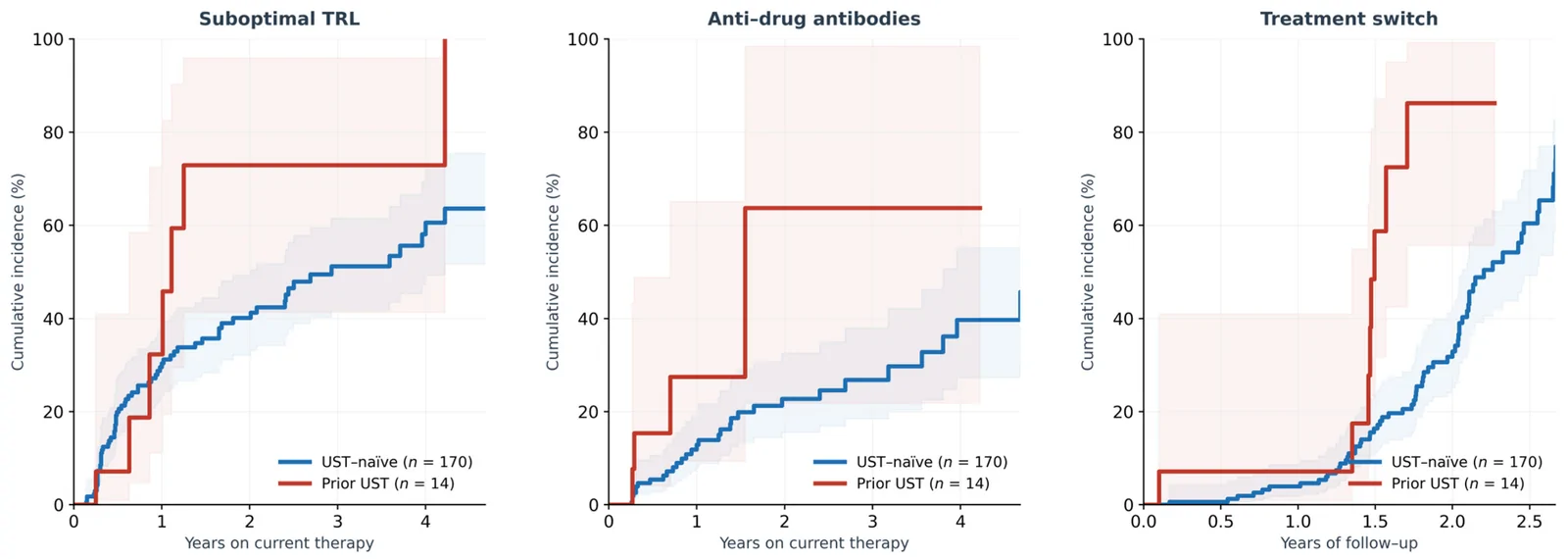
Kaplan–Meier curves stratified by prior ustekinumab (UST) exposure. Shaded bands represent 95% confidence intervals. The display complements the Cox-model hazard ratios by illustrating the timing and magnitude of the separation between patients with prior UST exposure and UST-naïve patients for each outcome. For TRL and ADA, the curves are exploratory descriptive displays based on the interval from treatment initiation to TDM sampling and should not be interpreted as estimates of the true timing or cumulative incidence of onset, because each outcome was assessed at a single TDM visit.

Across all three Cox models, prior UST exposure was consistently associated with adverse outcomes. UC subtype showed a similar, though more variable, pattern of higher estimated hazard. Kaplan–Meier curves stratified by prior UST exposure are shown in Figure 5. For treatment switching, the prior-UST group diverged from the group without prior UST exposure early in the course of current advanced-line therapy, and this divergence persisted throughout follow-up. For TRL and ADA, these curves provide exploratory descriptive displays across the observed treatment-to-TDM sampling intervals and should not be interpreted as estimates of the timing or cumulative incidence of onset.

### 3.6. Sensitivity Analyses

Proportional hazards assumption. Schoenfeld residual tests confirmed that the proportional hazards assumption was met for all covariates in the suboptimal TRL model (*p* = 0.118–0.892) and in the ADA model (*p* = 0.246–0.988). In the treatment-switch model, the assumption was violated for UC subtype (*p* = 0.002), age at disease onset (*p* = 0.003), concomitant immunosuppressant (*p* = 0.004) and current VDZ treatment (*p* = 0.019); the hazard ratios from this model therefore represent average effects over the follow-up period and should not be read as constant over time. Time-varying coefficient models were not pursued because the number of events (77) was too small to estimate them reliably.

Disease activity and inflammatory burden. Remission/relapse status at TDM was undocumented in 24 patients (13%). Each model was therefore refitted in the patients with complete activity data; the analysed samples comprised 160 patients (67 events) for the TRL model, 111 patients (26 events) for the ADA model and 160 patients (68 events) for the treatment-switch model. Three associations were unchanged (prior UST treatment HR = 3.58, 95% CI = 1.29–9.97, *p* = 0.015; UC subtype HR = 2.67, 95% CI = 1.34–5.31, *p* = 0.005; duration of prior biologic therapy HR = 0.90, 95% CI = 0.83–0.98, *p* = 0.015), whereas the association with current VDZ therapy became borderline (HR = 0.48, 95% CI = 0.22–1.06, *p* = 0.070). Prior UST exposure also remained associated with treatment switching (HR = 12.92, *p* < 0.001) and with ADA presence (HR = 5.68, *p* = 0.010) in this subgroup. Because relapse status and CRP are measured at the time of TDM and cannot serve as predictors of an earlier, unobserved event, adjustment for inflammatory burden was performed in the cross-sectional logistic framework, in which the outcome and inflammatory status are contemporaneous. With relapse status included (N = 160), none of the principal factors reached significance (prior UST OR = 1.50, 95% CI = 0.30–7.50, *p* = 0.622; UC subtype OR = 1.61, 95% CI = 0.63–4.07, *p* = 0.320; relapse OR = 1.78, 95% CI = 0.88–3.60, *p* = 0.109). After additionally including CRP (N = 148), CRP was the only factor independently associated with suboptimal TRL (OR = 1.04 per unit, 95% CI = 1.01–1.07, *p* = 0.008). Albumin was not included because measurements were available only in 142 of the 184 patients.

Analytical framework for TRL and ADA. Because TRL and ADA status were each derived from a single TDM measurement, the analysis time in the corresponding Cox models is the interval between initiation of therapy and sampling rather than an observed event time; these analyses are therefore reported as exploratory and were repeated in a cross-sectional framework that matches the measured status. In multivariable logistic models with time on therapy entered as a covariate, the duration of prior biologic therapy remained associated with suboptimal TRL (OR = 0.91, 95% CI = 0.83–0.99, *p* = 0.038), whereas prior UST treatment (OR = 1.98, 95% CI = 0.47–8.24, *p* = 0.349), UC subtype (OR = 1.66, 95% CI = 0.68–4.07, *p* = 0.267) and current VDZ therapy (OR = 0.91, 95% CI = 0.33–2.49, *p* = 0.847) were not. For ADA presence, prior UST treatment remained significantly associated in the logistic model (OR = 6.87, 95% CI = 1.30–36.28, *p* = 0.023). Sampling occurred earlier in patients with prior UST exposure (median 34.6 weeks) than in those without prior VDZ or UST exposure (52.5 weeks), whereas sampling time did not differ between UST- and VDZ-treated patients (48.6 vs. 54.1 weeks, *p* = 0.284). The association between prior UST exposure and ADA presence was therefore reproduced in both frameworks, whereas the TRL associations with prior UST exposure, UC subtype and current VDZ therapy were significant only in the time-to-detection analysis and are accordingly interpreted as hypothesis-generating.

Model stability and pooled populations. Because prior biologic exposure and current therapy were partly structurally linked, their distribution was examined directly. Among the 14 patients with prior UST exposure, all 14 received VDZ as their current therapy, of whom 7 had suboptimal TRL. Among the 30 patients with prior VDZ exposure, all received UST, of whom 12 had suboptimal TRL. Among the 140 patients without prior VDZ or UST exposure, 74 received UST, of whom 29 had suboptimal TRL and 66 received VDZ, of whom 31 had suboptimal TRL.

Collinearity diagnostics for the TRL model did not indicate substantial multicollinearity; variance inflation factors (for IBD subtype, duration of prior biologic therapy, age at disease onset, gender, prior UST exposure, prior VDZ exposure, and current VDZ therapy) ranged from 1.00 to 2.55, with all values below the conventional threshold of 5.

ADA measurements were available for all 184 patients; the smaller sample size of the ADA model (N = 122) resulted from missing documentation of prior immunosuppressant therapy, which was included as a covariate. Patients with and without this information did not differ in ADA positivity or in the proportion with suboptimal TRL.

As a sensitivity analysis, we refitted a more parsimonious ADA model without prior immunosuppressant therapy, allowing inclusion of 184 patients and 39 events. In this model, the association between prior UST exposure and detectable ADA was attenuated and no longer reached statistical significance (HR = 2.72, 95% CI = 0.90–8.18, *p* = 0.075); ridge-penalised refitting of the original model likewise attenuated the estimate (HR = 3.95, 95% CI = 1.28–12.22, *p* = 0.017). These analyses indicate that the direction of the association was preserved, but its magnitude and statistical significance varied according to model specification. Finally, interaction terms between prior UST exposure and age group (childhood-onset disease, interaction HR = 1.09, *p* = 0.941) and between prior UST exposure and current paediatric status at TDM (*p* = 0.996) showed no evidence of effect modification, which did not suggest a clear difference between paediatric and adult patients; however, these analyses had limited power because of the small prior-UST subgroup.

## 4. Discussion

In this multicentre retrospective cohort of paediatric and adult patients with IBD receiving VDZ or UST, we investigated real-world factors associated with suboptimal TRL and ADA presence using cross-sectional TDM data. Most patients were in remission or had mild disease activity. While VDZ and UST have been compared extensively in patients previously exposed to anti-TNF therapy [32,33,34,35,36], their role as third- or fourth-line agents has been investigated less thoroughly. Although a few studies have examined outcomes in patients with CD who failed prior biologic therapies [37,38,39,40], our cohort–comprising 184 patients from three IBD centres, including both CD and UC, with a substantial proportion having received at least two prior biologic agents–represents a broader real-world investigation in this setting.

Our Cox proportional hazards model identified prior UST exposure as being associated with an approximately fourfold higher hazard of suboptimal TRL. However, this finding should be interpreted cautiously because the prior-UST subgroup was small and all patients with prior UST exposure subsequently received VDZ. Several multicentre retrospective studies found no significant difference between VDZ and UST when used as third-line therapies in patients with CD [37,38,39,40]. One retrospective study reported that UST was more likely to achieve clinical remission at 14 weeks but observed no significant difference in efficacy at 52 weeks [32].

In addition to our findings, several retrospective cohort studies found no association between prior VDZ exposure and UST TRLs. In UC patients, anti-TNF therapy and UST showed similar effectiveness following VDZ failure [40]. In CD patients, prior VDZ use was associated with a lower likelihood of achieving endoscopic remission and contributed to LOR with UST [33,41]. The observed association between prior UST exposure and TRL in our study may reflect differences in the mechanisms of action between UST and VDZ or a more severe disease course in these patients.

In contrast, prior anti-TNF exposure was not associated with suboptimal TRL for either VDZ or UST in our study. The published data about the role of prior anti-TNF exposure are inconsistent. Several studies have reported that prior anti-TNF therapy worsens outcomes during VDZ or UST treatment [41,42,43]; conversely, some studies have found a beneficial effect [44] or no significant difference between biologic-naïve and biologic-exposed patients [45].

UC subtype was associated with an approximately threefold higher hazard of suboptimal TRL. Large-scale studies have reported more frequent and rapid biologic switching among patients with UC than among those with CD, which may reflect suboptimal TRLs. Notably, among patients treated with UST, switching is more frequent in UC than in CD, whereas VDZ appeared to be better tolerated for long-term use in UC [46,47]. Similarly, several studies have demonstrated that UST appears to be slightly more effective in achieving remission in CD than in UC [45,48,49], while VDZ maintains remission more effectively in UC [50,51,52]. Active UC is more likely to cause faecal loss of VDZ, contributing to suboptimal TRLs [23]. In our cross-sectional logistic framework, with relapse status included (N = 160), prior UST exposure and UC subtype were no longer significantly associated with suboptimal TRL (prior UST: OR = 1.50, 95% CI = 0.30–7.50, *p* = 0.622; UC subtype: OR = 1.61, 95% CI = 0.63–4.07, *p* = 0.320; relapse: OR = 1.78, 95% CI = 0.88–3.60, *p* = 0.109). When CRP was additionally included (N = 148), CRP was the only factor independently associated with suboptimal TRL (OR = 1.04 per unit, 95% CI = 1.01–1.07, *p* = 0.008), suggesting that inflammatory burden may partly account for the observed associations. The principal TRL associations observed in the exploratory Cox analysis were not reproduced in the cross-sectional analysis and were further attenuated after adjustment for inflammatory burden. Nonetheless, both agents remain effective across IBD subtypes [31,53].

Longer prior biologic treatment duration was associated with a lower hazard of suboptimal TRL. This finding is difficult to interpret definitively as it may reflect either a more refractory disease course requiring prolonged biologic exposure or a less active but longstanding disease with sustained therapeutic benefit. Biologic treatment duration in isolation is therefore unlikely to represent a reliable factor of VDZ or UST efficacy and should be interpreted as an exploratory association rather than an independent protective factor.

Although VDZ was associated with a lower hazard of suboptimal TRL in patients without prior UST exposure, this association likely reflects cohort composition and treatment-sequencing bias rather than a true drug-specific pharmacological advantage. In our cohort, 83.3% (55/66) of VDZ-treated patients without prior UST exposure had UC—a phenotype in which VDZ may be marginally more effective than UST. Furthermore, VDZ-treated patients underwent TDM at a median of 54.1 weeks after treatment initiation, compared with 48.6 weeks in other groups, introducing potential confounding by time on therapy.

Regarding ADA presence, previous development of ADAs against anti-TNF agents has not been shown to affect the presence of ADAs against UST or VDZ [34,54]. Both VDZ and UST are characterised by low immunogenicity, and when ADAs occur, they are typically transient and have little or no impact on treatment outcomes [55,56]. The factors associated with ADA presence during treatment with these agents remain largely undefined. Several studies have suggested that patients with a history of ADA development against prior biologics may be more susceptible to developing ADAs against subsequent agents [57]. A case–control study further demonstrated that patients who developed ADAs against prior anti-TNF therapy had a higher likelihood of developing ADAs against VDZ than against UST [57,58]. One study reported that VDZ retreatment increased the likelihood of ADA development [25].

We analysed ADA presence at a single time point rather than antibody development over time and found that prior UST exposure was the only significant factor associated with ADA presence in the primary Cox model, corresponding to an approximately 6.6-fold higher hazard of detectable ADA. However, this estimate was imprecise, with a wide confidence interval. In sensitivity analyses of the Cox model, the association became weaker and was no longer significant in the full cohort after exclusion of the incompletely documented immunosuppressant covariate. Given the variability of the estimates across model specifications, the limited number of ADA events, and the small prior-UST subgroup, this finding should be considered preliminary and requires validation in larger cohorts.

To safeguard the statistical integrity of the analysis, all *p*-values from the three Cox models were jointly adjusted for multiple comparisons using the Benjamini–Hochberg FDR procedure. Six of the seven originally significant associations retained significance, including prior UST exposure in all three models (q = 0.015 for each), UC subtype for suboptimal TRL (q = 0.015), duration of prior biologic therapy (q = 0.015) and current VDZ without prior UST exposure (q = 0.044). Only the association between UC subtype and treatment switch was no longer statistically significant after FDR adjustment (q = 0.092); therefore, this particular finding should be interpreted with caution.

## 5. Conclusions

Our findings highlight the increasing complexity of decisions regarding biologic switching as the spectrum of available biologic therapies expands and IBD manifests at progressively younger ages. Currently, no evidence-based guidelines address the sequencing of biologic therapies following failure of the first biologic treatment. Our study identified prior UST exposure and UC subtype as factors associated with suboptimal TRL, although the strength and statistical significance of these associations varied across analytical frameworks. Given the retrospective, cross-sectional design, the small size of the prior-UST subgroup, and the potential influence of disease behaviour on prior treatment duration, these findings should be considered hypothesis-generating and require validation in larger, independent prospective cohorts.

### Strengths and Limitations

The principal strength of this study lies in the broad and inclusive cohort design, which includes paediatric and adult patients, patients with both UC and CD, and a substantial proportion of patients with third-line or later-line biologic exposure. The main limitations are inherent in the retrospective and cross-sectional design. Our study had a relatively short follow-up duration, with a median follow-up of 20 months, which may be insufficient to fully assess the long-term impact of suboptimal TRL. The included TRL measurements were obtained at different time points during treatment, potentially contributing to heterogeneity in drug level assessments.

The primary ADA model was limited to 122 patients because prior immunosuppressant therapy was incompletely documented, and the relatively small number of ADA events constrained the number of covariates that could be included reliably in multivariable models. Owing to incomplete documentation in approximately 13% of patients, disease activity data at the TDM visit were unavailable, representing a potential source of bias. Quantitative drug concentrations were retrievable for only 130 of the 184 patients, whereas dichotomous TRL classification was available for the full cohort; therefore, TRL could not be analysed as a continuous outcome in the entire study population. Consequently, the robustness of the findings to alternative published TRL thresholds could not be assessed. Future studies with larger cohorts should also include age at disease onset and concomitant immunosuppressant treatment as candidate factors in ADA models.

Several candidate factors were tested simultaneously across three outcomes; although FDR adjustment was applied and confirmed the majority of the associations, the analyses remain exploratory and hypothesis-generating, and the individual associations require confirmation in independent cohorts. Because only 14 patients had prior UST exposure, estimates involving this subgroup, including the TRL, ADA, and treatment-switch associations, may be unstable and should be interpreted cautiously. Pooling paediatric and adult patients may also obscure age-specific differences, and the interaction analyses had limited power.

The reason for treatment switching was documented for only 21 of the 77 patients who switched therapy during follow-up. Consequently, a sensitivity analysis restricted to efficacy-related switches could not be performed reliably, as it would have been based on only approximately one quarter of the switching events. The associations with a higher or lower hazard of switching were derived solely from the Cox model estimates, and the treatment-switch outcome should be interpreted as an overall switching endpoint rather than a specific measure of treatment failure.

Finally, because TRL and ADA were each assessed at a single time point, the timing of the underlying events cannot be established exactly. For suboptimal TRL, the association with longer duration of prior biologic therapy was reproduced in the cross-sectional logistic model, whereas the associations with prior UST exposure, UC subtype, and current VDZ therapy were confined to the Cox analysis and should therefore be regarded as hypothesis-generating.

## Figures and Tables

**Table 1:** Clinical and treatment characteristics of patients receiving vedolizumab or ustekinumab for ulcerative colitis or Crohn’s disease.

	VDZ		UST		Total
Variable	CD (*n* = 24)	UC (*n* = 56)	CD (*n* = 86)	UC (*n* = 18)	*n* = 184
Sex (male/female)	11/13	26/30	41/45	8/10	86/98
Age at disease onset, median (IQR) (*n* = 184)	20.1 (16–27.2)	22.2 (14.5–31.4)	17.2 (13.0–26.4)	14.3 (11.9–30.0)	19.2 (13.1–27.7)
Childhood onset	29.2% (7/24)	35.7% (20/56)	48.8% (42/86)	50.0% (9/18)	42.4% (78/184)
Start of biologic treatment after diagnosis, years, median (IQR), (*n* = 184)	3.6 (0.6–10.3)	4.3 (1.7–10.1)	3.1 (0.7–7.9)	4.0 (1.2–8.8)	3.8 (0.9–9.0)
Prior anti-TNF	87.5% (21/24)	98.2% (55/56)	98.8% (85/86)	100% (18/18)	97.3% (179/184)
Prior VDZ/UST	54.2% (13/24)	1.8% (1/56)	19.8% (17/86)	72.2% (13/18)	23.9% (44/184)
Prior SMI	0% (0/24)	3.6% (2/56)	1.2% (1/86)	22.2% (4/18)	3.8% (7/184)
Duration of prior biologic therapy, years, median (IQR) (*n* = 184)	3.3 (1.1–8.5)	1.3 (0.6–3.6)	4.3 (1.1–7.0)	2.8 (0.9–5.8)	2.7 (0.9–5.8)
Relapse at switch	53.8% (7/13)	78.3% (36/46)	64.3% (36/56)	93.8% (15/16)	71.8% (94/131)
Interval from diagnosis to VDZ/UST initiation, years, median (IQR), (*n* = 184)	9.1 (4.2–15.0)	7.0 (3.8–16.4)	8.8 (4.2–12.9)	8.9 (5.2–12.1)	8.3 (3.9–14.4)
VDZ/UST treatment duration before measurement ^1^, weeks, median (IQR), (*n* = 184)	55.9 (26.2–112.5)	53.8 (23.9–147.1)	52.7 (24.1–115.9)	24.1 (14.9–44.8)	50.9 (22.8–116.3)
WBC, G/L, median (IQR), (*n* = 178)	7.9 (7–9.8)	7.7 (6.0–10.9)	8.4 (7.2–10.4)	7.3 (5.8–8.2)	8 (6.6–10.3)
Haemoglobin, g/L, median (IQR), (*n* = 176)	118.5 (105–139)	129 (120–142)	133 (119–146)	134 (113.3–139.8)	130 (116–142)
Platelets, G/L, median (IQR), (*n* = 175)	344.5 (289.5–464.3)	306 (270–380)	330 (273.8–385)	373 (285.5–501.3)	323.5 (275.3–406.8)
Albumin, g/L, median (IQR), (*n* = 142)	43 (40–44)	43 (41–46)	46 (42–48)	44 (41.5–46)	44 (41–47)
CRP, mg/L, median (IQR), (*n* = 170)	7.4 (0–17.2)	0 (0–9.2)	4.4 (0–18.3)	4.8 (0.9–10.4)	4 (0–15.3)
BMI, kg/m^2^, median (IQR), (*n* = 123)	22.0 (19.4–23.7)	20 (18.2–24.6)	21.5 (18.6–24.1)	22.6 (18.1–26.2)	21.4 (18.5–24.1)
PUCAI/PCDAI, median (IQR), (*n* = 64)	0 (0–20)	10 (5–20)	5 (0–17.5)	10 (7.5–22.5)	10 (0–20)
CDAI, median (IQR), (*n* = 40)	135 (93–153)	—	105 (45–158)	—	111 (51–157)
pMayo, median (IQR), (*n* = 38)	—	2 (1–4)	—	3 (3–4)	3 (1–4)
Disease localisation (CD: L1/L2/L3-4/NA; UC: E1/E2/E3-4/NA), (*n* = 146)	L1 12.5%/L2 29.2%/L3-4 41.7%/NA 16.7%	E1 10.7%/E2 28.6%/E3 39.3%/NA 21.4%	L1 15.1%/L2 19.8%/L3-4 41.9%/NA 23.2%	E1 11.1%/E2 11.1%/E3 66.7%/NA 11.1%	—
Upper GI tract involvement (CD only)	10.0% (2/20)	—	26.2% (16/61)	—	22.2% (18/81)
Perianal involvement (CD only)	5.0% (1/20)	—	15.0% (9/60)	—	12.5% (10/80)
Treatment intensification	15.8% (3/19)	8.9% (4/45)	19.7% (14/71)	12.5% (2/16)	15.2% (23/151)
Concomitant immunosuppressant	16.7% (3/18)	38.8% (19/49)	32.3% (21/65)	31.3% (5/16)	32.4% (48/148)
Concomitant steroid	33.3% (6/24)	42.3% (22/56)	23.1% (15/86)	31.3% (5/18)	31.8% (48/184)
5-ASA use	38.9% (7/24)	60.0% (30/56)	34.4% (21/86)	78.6% (11/18)	48.3% (69/184)
Surgery before TDM	33.3% (8/24)	8.9% (5/56)	33.7% (29/86)	11.1% (2/18)	23.9% (44/184)
Surgery after TDM	20.8% (5/24)	3.6% (2/56)	10.5% (9/86)	0% (0/18)	8.7% (16/184)
Suboptimal TRL ^2^	50.0% (12/24)	46.4% (26/56)	34.9% (30/86)	61.1% (11/18)	42.9% (79/184)
Antidrug antibody (ADA)	33.3% (8/24)	25.0% (14/56)	17.4% (15/86)	11.1% (2/18)	21.2% (39/184)
Relapse	35.0% (7/20)	41.2% (21/51)	32.4% (23/71)	72.2% (13/18)	40.0% (64/160)
Follow-up duration after TDM ^3^, months, median (IQR), (*n* = 184)	18.4 (17.2–25.2)	21.2 (16.4–25.4)	20.3 (15.4–25.0)	13.9 (6.0–20.4)	20.0 (15.3–24.9)
Biologic switch	50.0% (12/24)	55.4% (31/56)	32.6% (28/86)	33.3% (6/18)	41.8% (77/184)

Note: ^1^ VDZ/UST treatment duration was defined as the duration of the current biologic therapy from initiation until TDM. ^2^ Suboptimal TRLs were defined as VDZ < 15 µg/mL during induction and <12 µg/mL during maintenance, and UST < 3.5 µg/mL during induction and <1 µg/mL during maintenance. ^3^ Follow-up time was measured from TDM sampling to biologic switch or last follow-up. Data availability varied across variables because of the retrospective nature of the study. For categorical variables, data are presented as n/N, where n represents the number of patients with the characteristic and N the number of patients with available data for that variable. When data were complete, N corresponds to the total group size. For continuous variables, the number of available observations is reported in parentheses after the variable name. Abbreviations: IQR: interquartile range; VDZ: vedolizumab; UST: ustekinumab; CD: Crohn’s disease; UC: ulcerative colitis; anti-TNF: anti-tumour necrosis factor-alpha; SMI: small-molecule inhibitor; WBC: white blood cells; CRP: C-reactive protein; BMI: body mass index; PCDAI: Pediatric Crohn’s Disease Activity Index; PUCAI: Pediatric Ulcerative Colitis Activity Index; CDAI: Crohn’s Disease Activity Index; pMayo: partial Mayo score; 5-ASA: 5-aminosalicylates; TDM: therapeutic drug monitoring; TRL: trough level; CD localisation: L1: terminal ileum; L2: colonic; L3: ileocolonic; L4: isolated upper GI disease (modifier); UC extent: E1: proctitis; E2: left-sided colitis; E3: extensive colitis.

**Table 2:** Numerical results of the three Cox proportional hazards models. Hazard ratios (HR) with 95% confidence intervals (CI), unadjusted *p*-values and Benjamini–Hochberg false discovery rate (FDR) adjusted q-values are shown for every factor entered into each model. The FDR adjustment was applied jointly across all 19 tests performed in the three models. Continuous factors are expressed per unit increase (per year). Estimates correspond to those displayed in Figure 2, Figure 3 and Figure 4.

Factor	HR	95% CI	*p*-Value	FDR q	Reference Category
Model 1—Suboptimal TRL (*N* = 184, events = 79, χ^2^(7) = 22.51, *p* = 0.002)					
Prior UST treatment	4.13	1.58–10.81	0.004	0.015	Prior adalimumab/infliximab
UC subtype	2.83	1.39–5.76	0.004	0.015	CD
Prior VDZ treatment	0.65	0.30–1.41	0.274	0.473	Prior adalimumab/infliximab
VDZ (current therapy)	0.36	0.16–0.82	0.014	0.044	UST (current therapy)
Duration of prior biologic therapy, years	0.89	0.82–0.96	0.003	0.015	—
Age at disease onset, years	0.99	0.96–1.01	0.209	0.441	—
Gender, female	0.86	0.54–1.36	0.513	0.673	Male
Model 2—ADA presence (*N* = 122, events = 27)					
Prior UST treatment	6.59	1.95–22.23	0.002	0.015	Prior adalimumab/infliximab
Prior VDZ treatment	1.88	0.64–5.54	0.251	0.473	Prior adalimumab/infliximab
UC subtype	1.43	0.61–3.34	0.413	0.604	CD
Prior immunosuppressant	0.96	0.42–2.22	0.925	0.996	No immunosuppressant
Duration of prior biologic therapy, years	1.00	0.89–1.13	0.996	0.996	—
Model 3—Treatment switch during follow-up (*N* = 184, events = 77)					
Prior UST treatment	6.37	2.18–18.60	<0.001	0.015	Prior adalimumab/infliximab
UC subtype	2.27	1.06–4.85	0.034	0.092	CD
Concomitant immunosuppressant	1.01	0.63–1.62	0.968	0.996	None
Age at disease onset, years	1.01	0.98–1.03	0.567	0.673	—
Concomitant steroid	0.80	0.50–1.28	0.349	0.553	None
Prior VDZ treatment	0.78	0.36–1.72	0.542	0.673	Prior adalimumab/infliximab
VDZ (current therapy)	0.58	0.25–1.34	0.203	0.441	UST (current therapy)

Abbreviations: ADA: antidrug antibody, CD: Crohn’s disease, CI: confidence interval, FDR: false discovery rate, HR: hazard ratio, TRL: trough level, UC: ulcerative colitis, UST: ustekinumab, VDZ: vedolizumab.

## Data Availability

The datasets generated and/or analysed during the current study are not publicly available owing to patient privacy restrictions but are available from the corresponding author upon reasonable request.

## Abbreviations

The following abbreviations are used in this manuscript:

ADA	Antidrug antibody
BMI	Body mass index
CDAI	Crohn’s Disease Activity Index
CD	Crohn’s disease
CRP	C-reactive protein
EEN	Exclusive enteral nutrition
ELISA	Enzyme-linked immunosorbent assay
FDR	False discovery rate
HR	Hazard ratio
IBD	Inflammatory bowel disease
IBD-U	Inflammatory bowel disease unclassified
IQR	Interquartile range
LOR	Loss of response
OR	Odds ratio
PCDAI	Pediatric Crohn’s Disease Activity Index
pMayo	Partial Mayo score
PNR	Primary nonresponse
PUCAI	Pediatric Ulcerative Colitis Activity Index
SMI	Small-molecule inhibitor
TDM	Therapeutic drug monitoring
TNF-alpha	Tumour necrosis factor-alpha
TRL	Trough level
UC	Ulcerative colitis
UST	Ustekinumab
VDZ	Vedolizumab
WBC	White blood cells
